# Bias Toward Surgical Treatment of Acromioclavicular Injuries Exists on YouTube

**DOI:** 10.1016/j.asmr.2025.101170

**Published:** 2025-05-21

**Authors:** Eduard Van Eecke, Edward Mattelaer, Louis Thielman, Wouter Schroven, Michel P.J. van den Bekerom

**Affiliations:** a Department of Orthopaedic Surgery, Shoulder and Elbow Unit, OLVG, Amsterdam, The Netherlands; b Department of Orthopaedic Surgery, UZ Leuven, Leuven, Belgium; c Department of Orthopaedic Surgery, AZ Delta, Roeselare, Belgium; d Department of Human Movement Sciences, Faculty of Behavioral and Movement Sciences, Vrije Universiteit Amsterdam, Amsterdam Movement Sciences, Amsterdam, the Netherlands

## Abstract

**Purpose:**

To evaluate the quality and comprehensiveness of videos regarding acromioclavicular dislocation posted on the YouTube platform and to evaluate potential reinforcement of misinformation that may hinder proper management of these injuries.

**Methods:**

A YouTube search was performed in November 2024 using key words “acromioclavicular joint dislocation.” Videos were ranked on relevance and the first 50 videos that met inclusion criteria were analyzed by 2 reviewers. Video source, content type, time since upload, video duration, number of views, likes, subscribers and comments were recorded. Video educational quality was measured using the modified DISCERN, Journal of the American Medical Association (JAMA) score, Global Quality Score and Shoulder-Specific Score (SSS). Quality scores from different sources and content categories were compared using the Kruskal-Wallis test. Strength of relationship between variables was assessed using Spearman's rank correlation coefficient.

**Results:**

In total, 209,005 videos were identified of which the first 50 videos were analyzed. Mean mDISCERN, JAMA, GQS and SSS were 2.19, 2.13, 2.48, and 6.26, respectively. The most common uploader source were physicians (28%) and the most common content category was surgical management (32%). Videos uploaded by an academic source had significantly higher mDISCERN, JAMA, and SSS (*P* < .05). Other uploader sources did not show significant differences among each other. Quantitative video characteristics showed no significant correlation with quality scores, except the video duration. Finally, only 2 of 50 videos mention nonoperative treatment options for high-grade AC joint dislocations and only 3 of 50 videos refer to the lack of scientific evidence for operative treatment in these high-grade injuries.

**Conclusions:**

Current YouTube video content about AC dislocations has low overall quality, despite being mostly uploaded by physicians. It does not provide sufficient information and potentially reinforces misinformation that may downplay potential benefits of nonsurgical interventions.

**Clinical Relevance:**

Given the widespread use of YouTube by patients seeking medical information, evaluating the quality of this content is essential for surgeons to better understand and address the information their patients may encounter.

Acromioclavicular (AC) joint dislocation is a common shoulder injury and is reported to occur most in the young and athletic population.^1^ The incidence of acute AC joint disruptions accounts for 9% of all shoulder girdle injuries and up to 40% occur in elite athletes participating in competitive contact sports.^1^, ^2^, ^3^ Significant controversy remains regarding proper diagnosis and management of AC joint dislocations. Nonoperative management is accepted for low-grade injuries (Rockwood type I and II), whereas the optimal treatment for high-grade injuries is more controversial.^1^^,^^4^^,^^5^ Historically, these high-grade lesions were treated surgically, but recent studies have shown similar outcomes with nonoperative treatment.^6^, ^7^, ^8^, ^9^, ^10^ The current decision making process behind treatment in AC joint dislocations involves a complex interplay of various factors, including patient-specific considerations, injury characteristics, and surgeon experience. Therefore, the role of patient education and patient access to high-quality and timely information are extremely important to achieve informed decision making. Patient expectations, background knowledge, and attitude can be greatly influenced by what patients encounter online. Especially in this younger patient population, the use of the Internet to understand their health conditions is tremendously high.

YouTube is considered one of the most popular sources among Internet sites, with more than 2 billion users each month and 2 billion views each day reported.^11^ Beyond serving as a source of entertainment, YouTube also has emerged as a resource for health information.^11^ YouTube has the potential to be a valuable source of information for both patients and health care providers; however, the quality and reliability of videos found are variable. Finding medically related informative videos that offer accurate and useful guidance can be challenging resulting from the abundance of videos and unqualified sources that upload videos to YouTube. Unlike scientific literature, YouTube lacks a formal peer-review process, which raises concerns about the potential presence of unverified or misleading health information.

Therefore, The purposes of this study were to evaluate the quality and comprehensiveness of videos regarding AC dislocation posted on the YouTube platform and to evaluate potential reinforcement of misinformation that may hinder proper management of these injuries. We hypothesized that the quality of video content on AC joint dislocations would be low, be influenced by uploader source and content category, and videos might reinforce misinterpretations that may hinder proper management of these injuries.

## Methods

A search was conducted on November 11, 2024, using the key word “acromioclavicular joint dislocation” on the YouTube database. The search was performed with a cleared cache, cleared cookies in incognito browsing mode in Google Chrome. Although this reduces personalization on the basis of user history, it does not fully eliminate algorithmic influences such as location or regional trends, which may still affect the video rankings. Consistent with the standard user experience on YouTube, we maintained the default “relevance” filter for sorting the search results. This decision was made to closely mimic a typical user’s search behavior. Similar to previous YouTube-based studies in orthopaedic literature, the videos that appeared on the basis of the key word were sorted by relevance, and the first 50 videos were evaluated.^12^^,^^13^ These first 50 videos were recorded, evaluated for inclusion, and classified into categories according to their source and content.

Video source, time since upload, duration, and number of views and likes were recorded. View ratio (views per day) and number of likes per view were analyzed to evaluate viewer interaction parameters. Number of comments and subscribers were analyzed as well, similar to previous studies.^14^^,^^15^ English language was a prerequisite for inclusion of the video. The videos without audio, shorter than 30 seconds, repetitive, and containing irrelevant content, turned-off comments, or interactions by uploader and duplicated videos were excluded.

Similar to previously established methodology, the video sources/uploaders were categorized as follows: academic (pertaining to authors/uploaders affiliated with research groups or universities/colleges), physicians (independent physicians or physician groups without research or university/college affiliations), nonphysicians (health professionals other than licensed medical doctors), trainers, medical sources (content or animations from health websites), patients, and commercial sources studies.^14^^,^^15^ The content of each video was categorized as follows: disease-specific information, patient experience, surgical management, nonsurgical management, exercise training/rehabilitation, or advertisement.

Two reviewers (E.M. and L.T.) independently viewed and graded all 50 videos using the following scoring tools: the Modified DISCERN score, the Global Quality Score (GCS), the *Journal of the American Medical Association* (JAMA) Benchmark Score, and the Shoulder-Specific Score (SSS).^16^, ^17^, ^18^, ^19^ An average score from each tool was obtained.

**Fig 1:**
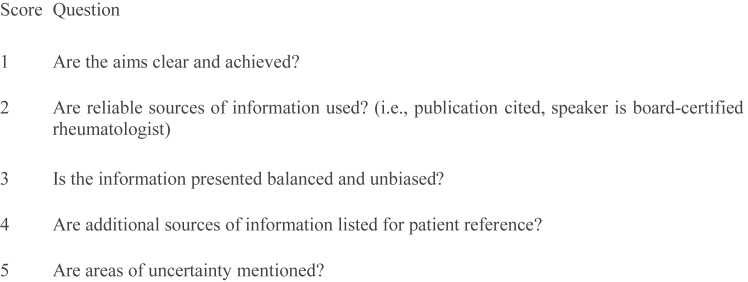
The modified DISCERN tool (1 point for every yes, 0 point for every no) (0 = low reliability, 5 = excellent reliability).

**Fig 2:**
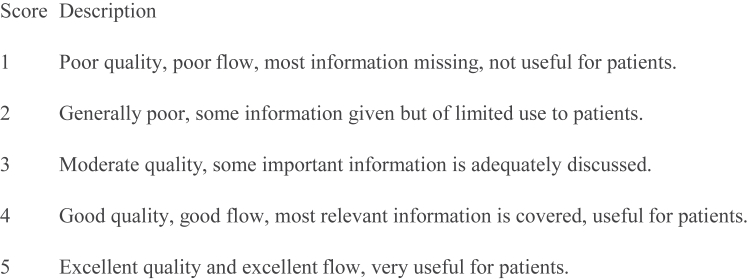
Global Quality Score (scored based on the following characteristics) (1 = poor quality, 5 = excellent quality.

**Fig 3:**
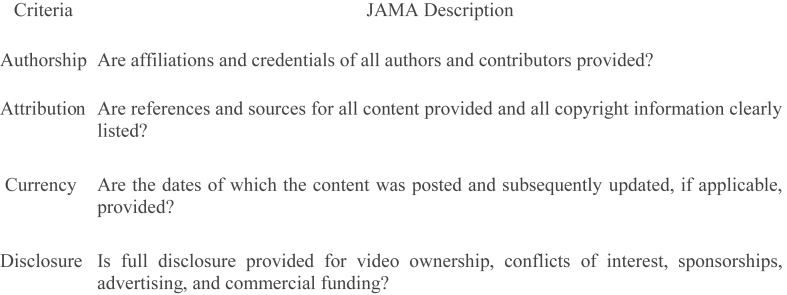
*Journal of American Medical Association* (JAMA) Benchmark Score (0 = poor authenticity/reliability, 4 = excellent authenticity/reliability.

Information quality scores were collected using a modified version of the DISCERN instrument, originally designed to assess the quality of written consumer health information regarding treatment options.^16^ The modified DISCERN tool allows for the evaluation of video content. In the modified DISCERN scale, each question was scored either 0 or 1 point. A total score of 5 indicates high reliability, whereas a 0-point shows low reliability in this scoring system (Fig 1). In addition, the GQS tool, consisting of 5 questions, was used to assess the overall quality, flow of information, and educational value of the videos.^18^ Each video is matched to 1 of the 5 descriptions, where greater scores (max of 5) indicate greater educational quality (Fig 2). Authenticity and accuracy of the videos were evaluated by the JAMA Benchmark Score.^17^ The JAMA Benchmark Score consists of scoring on the basis of 4 individual criteria: authorship, attribution, currency, and disclosure (Fig 3). One point is awarded for each criterion present in a video. Greater scores (max of 4) indicate greater video reliability.

**Fig 4:**
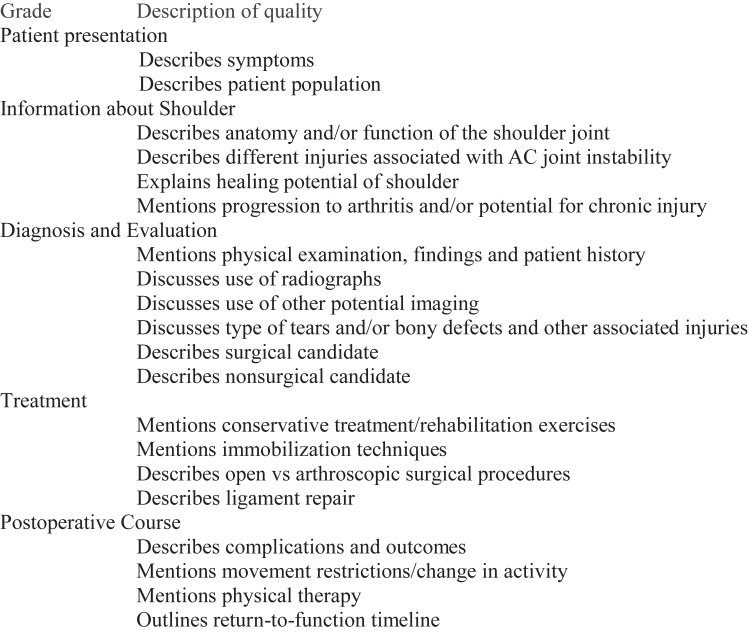
Modified Shoulder-Specific-Score (SSS).

To assess the quality of content specific to shoulder injuries, the SSS was used. Because no specific tool exists for assessing the quality of diagnostic and surgical information for AC joint dislocations, 2 minor adjustments were made to the SSS (Fig 4). This tool was developed by Etzel et al.^19^ The SSS grading criteria is composed of 20 items and evaluates information on common patient presentations and symptoms, anatomy of the shoulder, diagnosis and evaluation of shoulder pathologies, treatment options, and postoperative course and expectations. This tool was used to assess the level of potential reinforcement of unhelpful thinking in videos about AC joint dislocations. The current study was exempt from institutional review board approval because the study did not involve human subjects or animals.

### Statistical Analysis

All statistical tests were performed with IBM SPSS (version 22; IBM SPSS, Armonk, NY). The interclass correlation (ICC) (model: 2-way fixed, type: consistency) with 95% confidence intervals was used to analyze the 2 reviewer’s agreement on each scoring system. The scores of the 2 reviewers were averaged and used for further analysis. Descriptive statistics were used to quantify the video characteristics as well as the video reliability and quality scores. Continuous variables were presented as means and medians with standard deviations and ranges. Categorical variables were shown as relative frequencies with percentages. To compare the quality scores among videos from different uploader sources and content categories, the Kruskal-Wallis test was employed, a nonparametric method suitable for our data’s distribution characteristics. The Spearman correlation test was used to analyze the strength of the relationships between quantitative variables. Statistically significant results were indicated by a *P* value less than .05.

## Results

A total of 209,005 videos were identified using the key words “acromioclavicular joint dislocation.” Videos were sorted by relevance and the first 50 videos in our initial search that met the inclusion criteria were evaluated and analyzed. Descriptive data of the included videos are presented in Table 1.

**Table 1:** Descriptive of the Results (Characteristics, Viewer Interaction Parameters, Quality, and Reliability Scores of YouTube Videos About AC Joint Dislocation)

Characteristic	Mean	Median	Minimum	Maximum	SD
Video duration, min	7:42	4:55	0:46	36:21	8 min 28 s
Days since upload	1,812	1,594	304	4,466	1,261.95
Views	59,492	26,948	106	351,377	81,021.59
View ratio (views per day)	41	18.8	0.16	230.57	49.34
Likes	755	221.5	0	5300	1095.92
Comments	76	15.5	0	834	138.69
Subscribers	280,886	51,600	62	2,240,000	470,206.77
modified DISCERN	2.19	2	0	5	1.12
JAMA	2.13	2	1	4	0.74
GQS	2.48	2.5	1	4.5	0.87
SSS	6.26	5	0	16	4.34

AC, acromioclavicular; GQS, Global Quality Score; JAMA, *Journal of the American Medical Association*; SD, standard deviation; SSS, Shoulder-Specific-Score.

The ICC was performed for each scoring system and showed an agreement of 0.708 between the 2 reviewers for the mDISCERN, 0.483 for the JAMA score, 0.319 for the GQS, and 0.949 for the SSS. These results indicate poor reliability for the JAMA and GQS, moderate reliability for the mDISCERN, and excellent reliability for the SSS.

The averaged mDISCERN, JAMA, GQS, and SSS scores were 2.19, 2.13, 2.48, and 6.26, respectively. These results on the GQS indicate a generally poor-to-moderate educational quality and flow of health information. According to the mDISCERN score, the general reliability of the video content was low as well. The mean JAMA score illustrates questionable uploader sources with poor-to-moderate credibility. According to the mDISCERN score, 1 video was excellent, 6 were good, 17 were fair, 15 were poor, and 11 were very poor. GCS showed that 12 videos were good, 18 were fair, 15 were poor, and 5 were very poor. JAMA showed that 2 videos were good, 17 were fair, 24 were poor, and 7 were very poor. SSS showed that 2 videos were good, 9 were fair, 11 were poor, and 28 were very poor.

**Fig 5:**
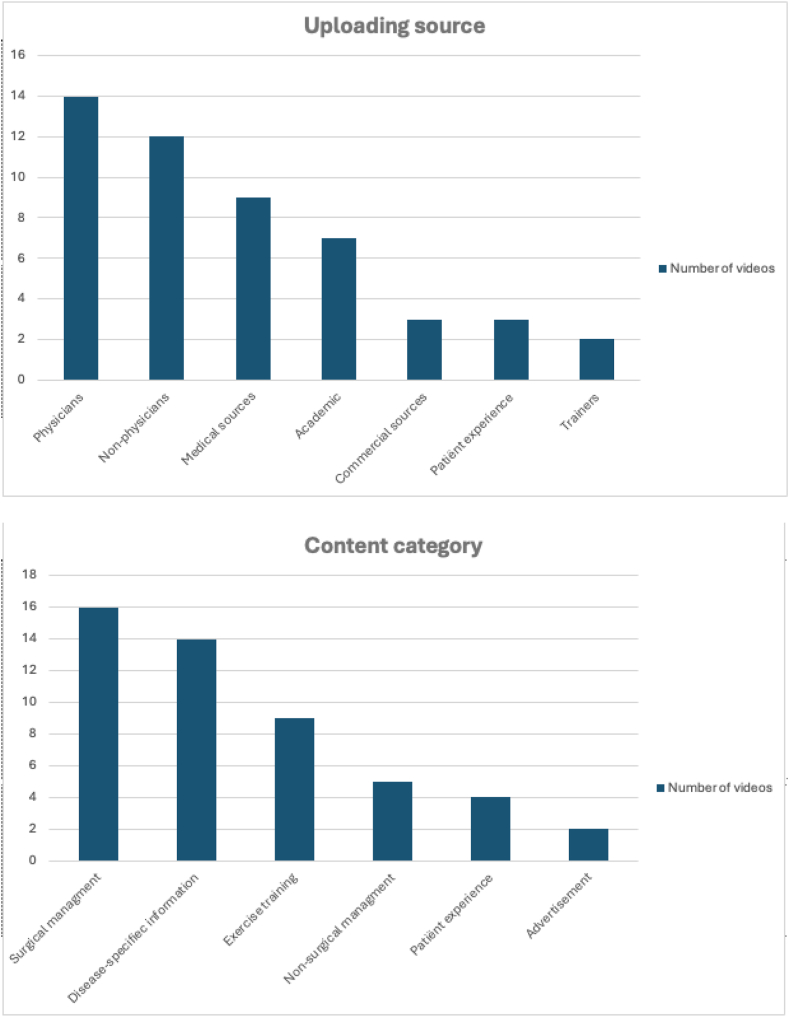
Videos per uploader source and content category.

The video uploading source were physicians in 14 of 50 (28%), nonphysicians in 12 of 50 (24%), medical sources in 9 of 50 (18%), academic in 7 of 50 (14%), patients in 3 of 50 (6%), commercial sources in 3 of 50 (6%), and trainers in 2 of 50 (4%). The content of the videos was categorized as surgical management in 16 of 50 (32%), disease-specific information in 14 of 50 (28%), exercise training/rehabilitation in 9 of 50 (18%), nonsurgical management in 5 of 50 (10%), patient experience in 4/50 (8%), and as advertisement in 2 of 50 (4%). An overview is shown in Figure 5.

**Fig 6:**
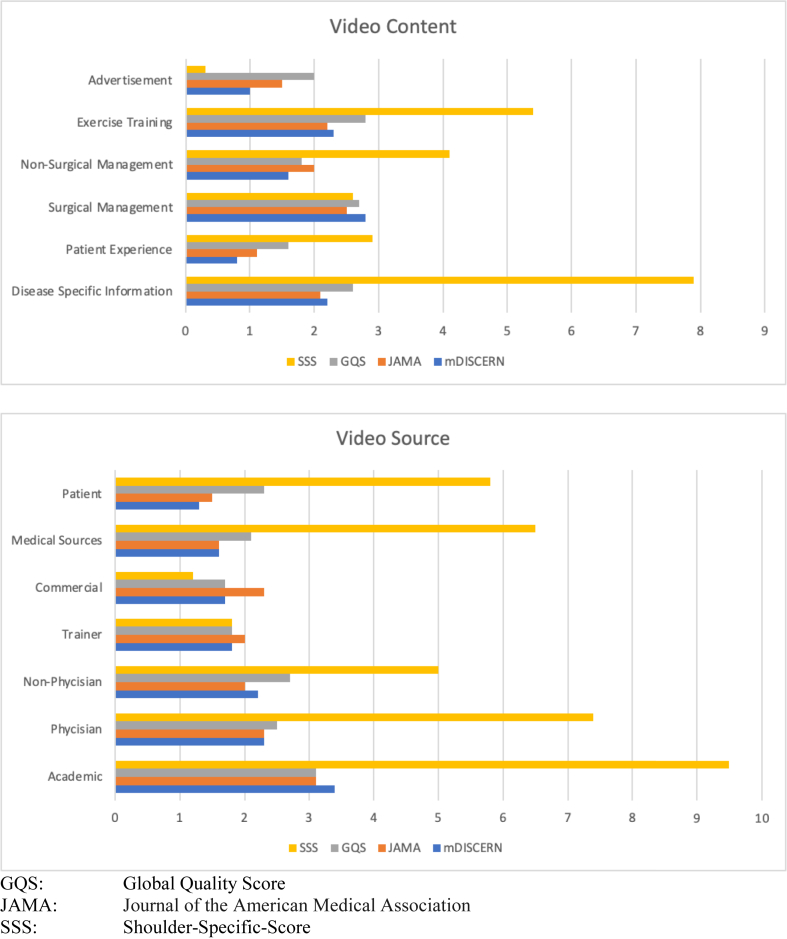
Quality scores per video content category and video source.

A Kruskal-Wallis test for non-normal data was used to determine whether the video reliability and the quality of educational content differed by uploader source and by content classification (Tables 2 and 3). An overview of quality scores per video content category and video source is shown in Figure 6. The mDISCERN score positively correlated with GQS, JAMA, and SSS (rho, 0.800, *P* < .001; rho, 0.693, *P* < .001; and rho, 0.617, *P* < .001, respectively).

**Table 2:** Comparison of Video Sources and Scores

Video Source	mDISCERN	JAMA	GQS	SSS
Academic (n = 7)	3.4 ± 0.9	3.1 ± 0.6	3.1 ± 0.8	9.5 ± 4.0
Physician (n = 14)	2.3 ± 1.1	2.3 ± 0.6	2.5 ± 0.8	7.4 ± 3.9
Nonphysician (n = 12)	2.2 ± 0.7	2.0 ± 0.6	2.7 ± 0.8	5 ± 2.7
Trainer (n = 2)	1.8 ± 0.4	2.0 ± 0	1.8 ± 0.4	1.8 ± 0.4
Commercial (n = 3)	1.7 ± 1.3	2.3 ± 0.6	1.7 ± 0.3	1.2 ± 1.6
Medical sources (n = 9)	1.6 ± 1.3	1.6 ± 0.6	2.1 ± 1.0	6.5 ± 6.3
Patient (n = 3)	1.3 ± 0.6	1.5 ± 0.9	2.3 ± 0.8	5.8 ± 1.9
*P* value	.036	.006	.077	.018

NOTE. Kruskal-Wallis test is presented as mean ± standard deviation.

GQS, Global Quality Score; JAMA, *Journal of the American Medical Association*; SSS, Shoulder-Specific-Score.

**Table 3:** Comparison of Video Content and Scores

Content	mDISCERN	JAMA	GQS	SSS
Disease-specific information (n = 14)	2.2 ± 1.1	2.1 ± 0.8	2.6 ± 0.9	7.9 ± 4.9
Patient experience (n = 4)	0.8 ± 0.5	1.1 ± 0.3	1.6 ± 0.6	2.9 ± 2.3
Surgical management (n = 16)	2.8 ± 1.06	2.5 ± 0.8	2.7 ± 0.8	2.6 ± 4.5
Nonsurgical management (n = 5)	1.6 ± 0.7	2 ± 0.7	1.8 ± 0.6	4.1 ± 2.4
Exercise training/rehabilitation (n = 9)	2.3 ± 1	2.2 ± 0.5	2.8 ± 1	5.4 ±2.7
Advertisement (n = 2)	1 ± 0.7	1.5 ± 0	2 ± 0	0.3 ± 0.4
*P* value	.009	.028	.056	.045

NOTE. Kruskal-Wallis test is presented as mean ± standard deviation.

GQS, Global Quality Score; JAMA, *Journal of the American Medical Association*; SSS, Shoulder-Specific-Score.

The number of views and duration since upload were negatively correlated with each score. View ratio was also negatively correlated with each score, except for JAMA. Video duration and subscribers were positively correlated with each score. The number of likes and comments were negatively correlated with the mDISCERN and GQS and weakly positively with JAMA and SSS (Table 3).

Finally, we specifically looked if 2 specific items were reported in the videos. Only 2/50 videos mention the nonoperative treatment option for high-grade AC joint dislocations (Rockwood grade IV and V). In addition, only 3 of 50 videos refer to the lack of scientific evidence for operative treatment in these high-grade injuries. In only 7 of 50 videos, it was explicitly stated with a disclaimer that you should contact your treating physician or orthopedic surgeon if you experience any complaints.

## Discussion

The main findings of this study are that the current YouTube video content has low overall quality, does not provide sufficient information on management of AC joint dislocations and potentially reinforces misinformation that may downplay the potential benefits of nonsurgical interventions. The mean JAMA, GQS, mDISCERN, and SSS scores were 2.19, 2.13, 2.48, and 6.26, respectively, revealing a poor reliability, accuracy, authenticity, and quality of shoulder-specific educational content of YouTube content on AC joint dislocations. A mean SSS score of 6.26 of 20 indicates that most videos fail to provide patients with sufficient/complete information about AC joint dislocations. Strikingly, even the traditionally more reputable sources (academic, physicians, medical sources) frequently missed more than one half of the important topics on AC joint dislocations.

These results are in accordance with previous studies, which have sought to evaluate the quality and content of orthopaedic topics on YouTube. Other orthopaedic-specific YouTube studies concerning topics as Dupuytren’s contracture, carpal tunnel syndrome, rotator cuff disease, reversed shoulder arthroplasty, shoulder instability and posterior cruciate ligament injury have all concluded that the quality and reliability of YouTube videos discussing these topics are strikingly low.^2^^,^^15^^,^^19^, ^20^, ^21^, ^22^, ^23^

The low ICC scores and lack of significance found with the GQS scores, as compared with the other scoring tools in this study, raises the question whether this tool is a useful/reliable instrument for quality assessment of patient educational video content. Spearman correlation, however, showed strong and very strong correlations between the different quality scores. This means that the same video was graded similar with each scoring tool.

The Kruskal-Wallis test in our study show that university channels/academic uploader sources have significantly better outcomes in quality scores compared with other uploader source types. In contrast, it is surprising that the “physicians” group, who actually present the largest uploader group, did not reveal better outcomes than other sources since they were expected to provide greater quality information than other sources, given the involved knowledge and expertise.

The quantitative variables shown in Table 4 showed no significant correlation with the quality scores and thus are not reliable parameters when searching for video quality. However, the duration of the videos was significantly correlated with the quality scores. We believe this can be explained by the fact that the longer a video is, the more boxes can be checked off in the quality scores. The duration since upload was correlated negatively with each score. Videos that were uploaded longer ago may reflect outdated information, production styles, or editing standards, especially in fast-moving niches like medicine.

**Table 4:** Correlations of Quantitative Variables and Scores

Content	mDISCERN (*P*; rho)	JAMA (p; rho)	GQS (*P*; rho)	SSS (*P*; rho)
Video duration, min	.001; 0.556	.001; 0.698	.001; 0.489	.001; 0.716
Days since upload	.299; −0.150	.645; −0.067	.089; −0.243	.189; −-0.195
Views	.116; −0.225	.823; −0.032	.153; −0.205	.713; −0.053
Likes	.410; −0.119	.456; 0.108	.407; −0.120	.943; 0.010
Comments	.432; −0.114	.561; 0.084	.372; −0.141	.975; 0.005
Subscribers	.248; 0.166	.260; 0.162	.051; 0.278	.672; 0.061
View ratio	.208; −0.181	.686; 0.059	.160; −0.202	.791; −0.038

NOTE. Values shown are *P* value; rho = Spearman’s rho.

GQS, Global Quality Score; JAMA, *Journal of the American Medical Association*; SSS, Shoulder-Specific-Score.

Another important finding in this study is the thorough focus on surgery. Surgery centrism in YouTube videos about AC joint dislocations can be problematic, as it perpetuates the misconception that all high-grade AC joint dislocations require surgical intervention. Only 2 of 50 videos mention nonoperative treatment as an option for high-grade injuries and 3 of 50 videos report about the lack of scientific evidence for surgical treatment. A major concern is the lack of comprehensive patient education regarding the indications for surgery, as well as an absence of information on recent guidelines and scientific research that inform treatment choices. This blanket approach and the overemphasis on surgical solutions and surgical techniques can reinforce misinformation and may inadvertently downplay the potential benefits of non-surgical interventions. As a result, viewers may receive incomplete or skewed information, which can lead to unnecessary anxiety and a misconception that surgery is the only viable option for high-grade AC joint dislocations. The gap in education about up-to-date evidence-based guidelines contributes to a growing misperception that surgery is the default treatment, when in fact, a more personalized, patient-centered approach is often appropriate.

YouTube, the second largest social media platform with more than 2 billion views per day, has increasingly become a source that patients use for medical information due to its easy access and format.^11^ However, it does not have a peer-review process for its videos, content is uploaded freely and is not required to meet certain regulations or scientific standards. The significance of source credibility, as highlighted by our findings, reinforces the need for health care providers to guide patients toward more reliable sources of health information. The observation that current YouTube video content about AC dislocations overemphasizes the role of operative treatment and disregards evidence that operative and nonoperative treatments have comparable outcomes suggests that these videos may be relatively promotional and self-serving. Patients should understand that YouTube videos may mostly be a form of marketing and seek out more balanced and dispassionate information. The ease of access and popularity of the YouTube platform could provide a unique opportunity for physicians and academic centers to produce quality educational content for their patients.

### Limitations

This study is not without limitations. First, only English-language videos were analyzed. Second, the fact that this study only represents the YouTube platform may limit generalization to all social media. Another limitation of the study is the subjective nature of the JAMA, GCS, mDISCERN, and SSS scoring tools. Furthermore, only the first 50 videos were analyzed in this study. Although this number represents a small proportion of YouTube libraries, previous studies showed that most of the internet users selected a search result within the first 3 pages.^18^ Next, video “quality” can encompass a variety of factors and not all of them may be addressed in the current study including video production, verbal bias and the accurate depiction of surgical footage. Finally, a single time point was used to evaluate the quality and reliability of the videos.

## Conclusions

Current YouTube video content about AC dislocations has low overall quality, despite being mostly uploaded by physicians. It does not provide sufficient information and potentially reinforces misinformation that may downplay potential benefits of nonsurgical interventions.

## Disclosures

The authors declare the following financial interests/personal relationships which may be considered as potential competing interests: M.v.d.B. reports grants for clinical and research fellowships supported by 10.13039/100009026Smith & Nephew. All other authors (E.P.C.V.E., E.M., L.T., W.S.) declare that they have no known competing financial interests or personal relationships that could have appeared to influence the work reported in this paper.
